# Bibliometric analysis of medicine – related publications on refugees, asylum-seekers, and internally displaced people: 2000 – 2015

**DOI:** 10.1186/s12914-017-0116-4

**Published:** 2017-03-20

**Authors:** Waleed M. Sweileh

**Affiliations:** Department of Physiology, Pharmacology, and Toxicology, College of Medicine and Health Sciences, An-Najah National University, Nablus, Palestine

**Keywords:** Refugees, Asylum seekers, Internally displaced people, Bibliometrics

## Abstract

**Background:**

Wars and violent domestic conflicts have forced millions of people to move outside their homes. Meeting the basic health needs of those people requires an understanding of research activity and research output on this topic. The objective of this study was to shed light on the quantity and impact of medicine – related publications on refugees, asylum seekers and internally displaced people (IDP).

**Method:**

Scopus database was used to retrieve required data. Specifically, the number of publications, top productive countries and institutions, highly cited articles, citation analysis, international collaboration, and journals involved in publishing articles on refugees, asylum seekers and IDP were reviewed and analyzed. The time span for the study was set from year 2000 to 2015.

**Results:**

Two thousands five hundred and thirty publications were retrieved. The *h*-index of retrieved articles was 64. A steep rise in number of publications was noticed after 2011. Top productive countries were the United States of America, Australia and the United Kingdom. The American public health institute (Centers for Disease Control and Prevention) and the United Nations refugee agency were among the top active organizations on this topic. Active journals in publishing on health of refugees, asylum seekers and IDP were those on mental health, psychology, public health and general medicine. Publications on Somali, Afghani, Iraqi, and Syrian refugees received a significant share of medicine-related publications. Analysis of publications based on region showed that publications on refugees from Middle East is rising sharply and is approaching those on African refugees.

**Conclusion:**

Bibliometric analysis reveals that research publications on refugees have been increasing in a dramatic way and articles are being published in journals with high impact factor and international reputation, not only in general medicine and public health, but also mental health and psychology journals. Analysis of publications related to refugees can be helpful to international health agencies and governments not only to document the psychological trauma of fled people, but also to identify best mental health programs to face the consequences of wars and aggression that led to this refugee crisis.

## Background

In the past few decades, wars and violent conflicts in several parts of the world have forced millions of people to move outside their homes [1]. This has led to the emergence of refugee camps and hundreds of thousands of people seeking asylum in different host countries. People in refugee camps or those waiting for asylum are living in harsh environmental and health conditions with minimum healthcare services that will ultimately increase their risk of having physical and mental diseases [2–4]. Refugees and asylum seekers are of different ethnicities and have a wide range of physical and mental health needs [5–7]. The Syrian crisis is a typical example of health challenges facing refugees themselves and host countries [8]. Reports on increasing prevalence of infectious diseases, psychiatric problems, cardio-metabolic and malnutrition among refugees have been published [9, 10]. One way to promote health among refugees and asylum seekers is by doing research on health needs of these people and providing care and services accordingly [11]. Research on this topic indicated that mental health services to refugees and migrants are of top priority [12–15].

A recent report titled “World at War” published by United Nations High Commissioner for Refugees (UNHCR) stated that: “By end-2014, 59.5 million individuals were forcibly displaced worldwide as a result of persecution, conflict, generalized violence, or human rights violations” [16]. The report also stated that in year 2014, the number of individuals forced to leave their homes per day increased four-fold in four years and reached an average of 42,500 individuals per day. More than half (53%) of all refugees worldwide came from just three countries: the Syrian Arab Republic (3.88 million), Afghanistan (2.59 million), and Somalia (1.11 million). Unfortunately, women, children and elderly constitute a large percentage of displaced people who need continuous psychosocial support.

Bibliometric analysis is a mathematical tool used to assess the quantity and impact of research, publications, and extent of success accomplished on a certain topic [17, 18]. Bibliometric analysis has been applied to various medical disciplines in order to assess research trends and suggest future research ideas [19–24]. Bibliometric analysis is important for young researchers to help them identify research leaders and issues of health importance. Bibliometric analysis allows health policymakers to implement preventive measures, if, for example, bibliometric analysis indicates a rising number of articles on a certain issue or geographical location. Furthermore, assessment of publications will help international agencies to program international health aids and support in a more effective way.

Review articles on various health aspects of refugees have been published [25–30]. However, no bibliometric studies or assessment of research output on refugees, asylum seekers and internally displaced people (IDP) from a medical point of view have been published. Therefore, we carried out this bibliometric study to assess the growth of publications, active countries and institutions, highly cited articles, citation analysis, international collaboration, journals involved in publishing articles on refugees, asylum seekers and IDP to determine if it is indeed recognized as a growing health problem from a public and mental health aspects.

## Methods

For the purpose of this study, articles on refugees, asylum seekers and IDP published in journals categorized under the subject “Medicine” in Scopus database were retrieved and analyzed. The steps carried out to retrieve the required data were as follows: (1) the words “refugee” OR “asylum seeker” OR “displaced people” were entered in Scopus search engine as title search; (2) the time span was set from year 2000 to 2015; (3) the retrieved documents were refined by limiting search to documents categorized under the subject “medicine”; (4) the refined documents were further limited to include documents published in peer review journals only and to exclude books and book chapters; (5) any journal document classified as erratum (correction) was also excluded since it does not represent a new document; and finally (6) retrieved data were exported to Microsoft Excel or Statistical Package for Social Sciences for data and graphical presentation.

The search strategy in this study was based on searching for the required terms in article title to maximize accuracy and minimize false positive results. The words used in this study (refugee, asylum, displaced) are not unique ones and using a loose search strategy will retrieve high percentage of false results. The validity of the search strategy implemented was confirmed by manually reviewing at least 10% of retrieved articles to make sure that all retrieved articled were within the scope of the study. The selection of articles for validity check was based on either highly cited articles or zero article cited. Therefore, the validity check included old and newly published articles. The manual review of 10% of selected documents showed that all retrieved documents were within the scope of the study. The results pertaining to various ethnicities of refugees were obtained by searching the retrieved data for the particular ethnic group. For example, to obtain documents on Syrian refugees within the retrieved documents, we searched within the retrieved documents for the word “Syria*”. The asterisk is used as a wild card to retrieve documents with the word Syria or Syrian. The same strategy applies to Iraqi, Somali and other ethnicities.

The selection of Scopus database to perform this study was based on the advantages it has over other databases like Web of Science, Medline or Google scholar [31]. Scopus offers about 20% more coverage than Web of Science, whereas Google Scholar offers results of inconsistent accuracy [31]. Scopus has several features that allows detailed bibliographic analysis. Such features include country, institution, and author analysis from a quantitative and qualitative points of view. Scopus has the ability to rank countries based on the number of publications. Scopus counts the number of publications based on the country affiliation of authors in the article regardless of the position of the author. For example, when Scopus indicates that a country X has 100 publication, then this means that country X appears as a country affiliation in 100 publications regardless of the position of the author affiliated with country X. If a certain publication has two authors, one with country affiliation of X and the second one with a country affiliation of Y, then this publication will be counted twice, one for country X and one for country Y. Therefore the total sum of contributions by all countries will be greater than 100% because of the overlap. Publications with multiple authors of the same country affiliation is counted once. The same applies to ranking institutions and authors.

The impact of publications in any field could be assessed indirectly using indicators such as average number of citations per article, Hirsch-index (*h*-index), percentage of highly cited articles, and impact factor (IF) of journals publishing the documents of interest. H-index has been developed to assess productivity and citation impact of individual researchers [32]. However, the use of *h*-index has been extended to measure the productivity and citation impact of countries and academic institutions [32]. For the purpose of this study, we considered an article with a minimum of 30 citations to be highly cited. In this study, *h*-index for retrieved data was obtained from Scopus database while IF was obtained from Journal Citation Report 2015 published by Thompson Reuters [33]. To visualize country co-authorships (international collaboration), VOSviewer mapping technique was used [34].

No ethical approval for this study was required by university IRB since no human subjects or data were collected. All data analysis was carried out on September 02^nd^, 2016 to avoid the dynamic changes of citations from one day to another.

## Results

### General information

A total of 2530 journal articles were retrieved. The total citations assigned for retrieved documents was 29,174, an average of 19.07 citations per article. The *h*-index of retrieved articles was 64 which means that there are at least 64 articles with a minimum of 64 citations. Citation analysis showed that 1931 (76.3%) articles were cited at least once while the remaining articles were not cited at all. Approximately 10% (261) of retrieved articles were highly cited (i.e. have a minimum of 30 citations).

**Fig. 1:**
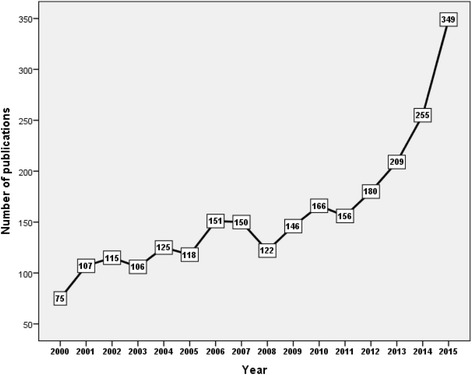
Growth of medicine – related publications on refugees, asylum seekers, and internally displaced people (2000 – 2015)

Retrieved articles were written in 24 different languages. English (2,343; 92.6%) was the most common. A total of 97 different countries participated in the production of retrieved articles. Types of retrieved articles were diverse, but research articles (1,845; 72.9%) were most common followed by review articles (214; 8.5%), letters (141, 5.6%), notes (129, 5.1%), editorials (84, 3.3%), short surveys (57, 2.3%), articles in press (36, 1.4%), and conference papers published in peer reviewed journals (24, 1.0%). Retrieved articles showed an increasing growth pattern with time. The highest number of publications was recorded in 2015 with 350 articles compared to 75 articles published in year 2000. Figure 1 shows the growth of publications from year 2000 to year 2015. The steepest growth pattern was seen after year 2011. The growth pattern from year 2000 to year 2010 was slow and sometimes steady.

### Journals, countries, institutions

A total of 19 peer reviewed journals had published at least 20 articles each. The total number of articles published by these journals was 683 (27.0%). The most active journals on this topic and ranked first were *Journal of Immigrant and Minority Health* and *Lancet* with 97 (3.8%) publications for each. Most of the journals in the list were in general medicine, public health, and mental health. Table 1 shows the list of journals with a minimum publications of 20 articles. Most of the journals in the list had an IF and some of them had high IF.

**Table 1:** List of journals that had published at least 20 articles on refugees, asylum seekers, and internally displaced people

SCR	Journal	N	%	IF	SCR	Journal	N	%	IF
1^st^	*Journal of Immigrant and Minority Health*	97	3.8	1.482	11^th^	*British Medical Journal*	25	1.0	19.967
1^st^	*Lancet*	97	3.8	45.217	11^th^	*Conflict and Health*	25	1.0	N/A
3^rd^	*Medical Journal of Australia*	45	1.8	4.089	13^th^	*Torture Journal*	23	0.9	N/A
3^rd^	*Transcultural Psychiatry*	45	1.8	2.114	14^th^	*American Journal of Public Health*	22	0.9	4.552
5^th^	*BMJ Clinical Research Ed*	41	1.6	19.967	14^th^	*American Journal of Tropical Medicine and Hygiene*	22	0.9	1.67
6^th^	*Journal of Nervous and Mental Disease*	40	1.6	1.688	14^th^	*BMJ Online*	22	0.9	1.53
7^th^	*Journal of Traumatic Stress*	38	1.5	2.360	17^th^	*Australian and New Zealand Journal of Public Health*	21	0.8	1.628
8^th^	*BMC Public Health*	27	1.1	2.264	17^th^	*Lakartidningen*	21	0.8	0.05
9^th^	*Plos One*	26	1.0	3.234	19^th^	*Journal of Paediatrics and Child Health*	20	0.8	1.00
9^th^	*Social Science and Medicine*	26	1.0	2.814					

*SCR* Standard competition ranking. Equal countries were given the same ranking number, and then a gap is left in the ranking numbers, *IF* impact factor, *N/A* not applicable

**Fig. 2:**
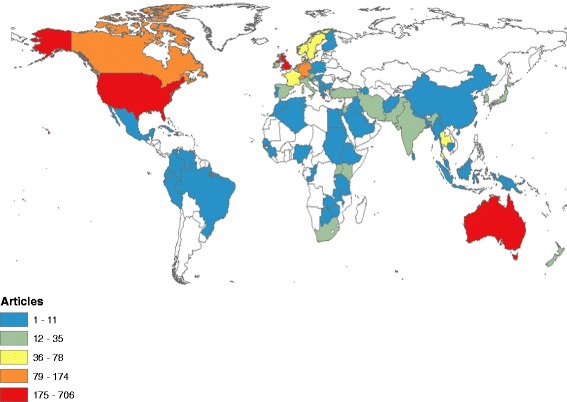
Geographical distributions of medicine – related publications on refugees

**Fig. 3:**
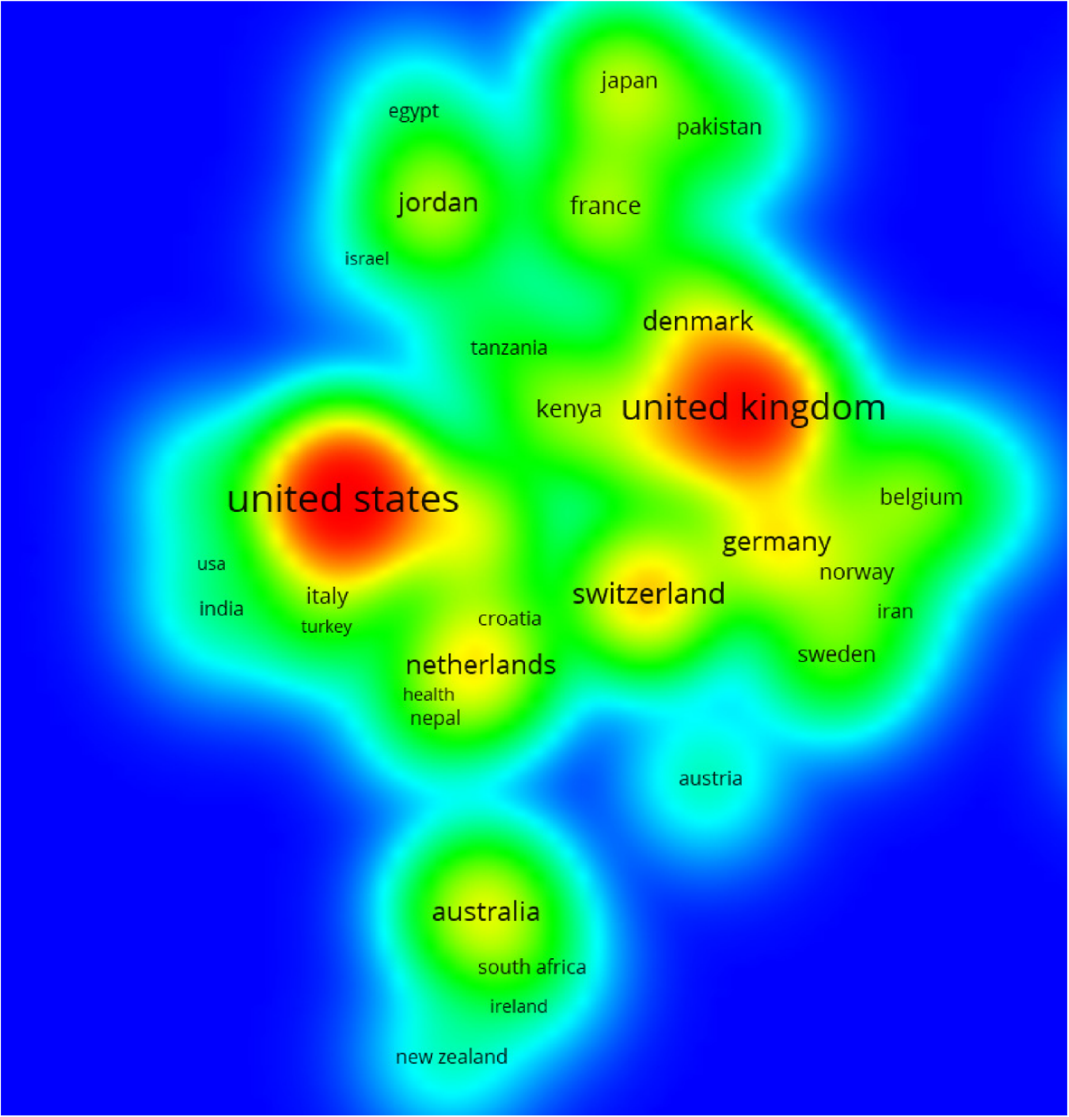
Density visualization map for country co-authorship (international collaboration)

Research output on medicine – related problems of refugees was presented in a geographical map using ArcMap10.1 software (Fig. 2). In the map, dark red countries represent countries with the highest productivity of medicine – related publications about refugees. Table 2 shows a list of countries with a minimum of 20 publications. A total of 20 different countries had published at least 20 articles each. The total number of articles published by these 20 countries was 2349 (92.9%). The United States of America (USA) contributed most (700, 27.8%) followed by Australia (13.2%) and the United Kingdom (UK) (323, 12.%). Country co-authorship analysis (international collaboration) showed that the USA had the greatest international collaboration in this topic followed by the UK (Fig. 3). The extent of international collaboration in the VOSviewer density visualization map is judged by the density of red color around the specified country.

**Table 2:** List of countries that had published a minimum of 20 articles on refugees, asylum seekers and internally displaced people

SCR	Country	N	%	SCR	Country	N	%
1^st^	USA	700	27.8	11^th^	Norway	47	1.9
2^nd^	Australia	335	13.2	12^th^	Thailand	40	1.6
3^rd^	UK	323	12.8	13^th^	Italy	35	1.4
4^th^	Canada	175	6.9	14^th^	Jordan	34	1.3
5^th^	Netherlands	111	4.4	15^th^	Belgium	30	1.2
6^th^	Switzerland	105	4.2	16^th^	Pakistan	30	1.2
7^th^	Germany	95	3.8	17^th^	Kenya	28	1.1
8^th^	Sweden	76	3.0	18^th^	New Zealand	24	1.0
9^th^	Denmark	71	2.8	19^th^	Lebanon	22	0.9
10^th^	France	47	1.9	20^th^	South Korea	20	0.8

*SCR* Standard competition ranking. Equal countries were given the same ranking number, and then a gap is left in the ranking numbers, *USA* United States of America, *UK* United Kingdom

A total of 23 institutions have published at least 20 articles each. The list of institutions is shown in Table 3. The total number of articles published by the institutions in the list was 775 (30.6%). The *Centers for Disease Control and Prevention* (CDC) was the most productive with 73 (2.9%) publications followed by *University of New South Wales* (Australia) with 66 (2.6%) publications. The *United Nations High Commissioner for Refugees (UNHCR)* ranked 10^th^ with a total publication of 36 (1.4%) articles. Eight of the active institutions are in the USA while six are in Australia and three are in the UK.

**Table 3:** List of active institutions/organizations that had published at least 20 articles on refugees, asylum seekers and internally displace people

SCR		N (%)	Affiliation	SCR		N (%)	Affiliation
1^st^	*Centers for Disease Control and Prevention*	73 (2.9)	USA	13^th^	*University of Western Australia*	27 (1.1)	Australia
2^nd^	*University of New South Wales UNSW Australia*	66 (2.6)	Australia	14^th^	*Arbour Counseling Services*	26 (1.0)	USA
3^rd^	*University of Melbourne*	47 (1.9)	Australia	14^th^	*King’s College London*	26 (1.0)	UK
4^th^	*Monash University*	42 (1.7)	Australia	16^th^	*Mahidol University*	25 (1.0)	Thailand
4^th^	*London School of Hygiene & Tropical Medicine*	42 (1.7)	UK	17^th^	*University of Oxford*	24 (1.0)	UK
6^th^	*University of Toronto*	41 (1.6)	Canada	18^th^	*University of Adelaide*	22 (0.9)	Australia
7^th^	*University of Minnesota Twin Cities*	38 (1.5)	USA	18^th^	*Karolinska Institutet*	22 (0.9)	Sweden
7^th^	*Massachusetts General Hospital*	38 (1.5)	USA	18^th^	*University of Illinois at Chicago*	22 (0.9)	USA
9^th^	*McGill University*	37 (1.5)	Canada	21^st^	*Harvard Medical School*	21 (0.8)	USA
10^th^	*United Nations High Commissioner for Refugees*	36 (1.4)	UN	22^nd^	*La Trobe University*	20 (0.8)	Australia
11^th^	*Johns Hopkins Bloomberg School of Public Health*	32 (1.3)	USA	22^nd^	*Shoklo Malaria Research Unit*	20 (0.8)	Thailand
12^th^	*Boston University*	29 (1.2)	USA				

*SCR* Standard competition ranking. Equal countries were given the same ranking number, and then a gap is left in the ranking numbers, *USA* United States of America, *UK* United Kingdom, *UN* United Nations

### Top cited articles

The top 20 cited articles on refugees, asylum seekers and IDP are shown in Table 4. The highest number of citations obtained was 458 for the article titled *“Prevalence of serious mental disorder in 7000 refugees resettled in western countries: A systematic review”.* The top list included eight review articles while the remaining 12 articles were research articles. At least 16 articles in the top 20 list discussed mental and psychological health of refugees while the remaining four articles were in general medicine and public health. Journals in the field of psychiatry and mental health dominated the top 20 list of highly cited articles. However, two articles in the top 20 list were published in *Lancet* while four were published in *Journal of the American Medical Association*.

**Table 4:** Top 20 cited articles

SCR	Title	Number of citations	Type of article	Reference
1^st^	Prevalence of serious mental disorder in 7000 refugees resettled in western countries: A systematic review	458	**Review**	[54]
2^nd^	Predisplacement and postdisplacement factors associated with mental health of refugees and internally displaced persons: A meta-analysis	377	**Review**	[55]
3^rd^	Mental health of Cambodian refugees 2 decades after resettlement in the United States	256	Article	[56]
4^th^	A comparison of narrative exposure therapy, supportive counseling, and psychoeducation for treating posttraumatic stress disorder in an African refugee settlement	240	Article	[57]
5^th^	Measuring trauma and health status in refugees: A critical review	227	**Review**	[58]
6^th^	Longitudinal study of psychiatric symptoms, disability, mortality, and emigration among Bosnian refugees	202	Article	[59]
7^th^	Review of child and adolescent refugee mental health	195	**Review**	[60]
8^th^	Long-term effect of psychological trauma on the mental health of Vietnamese refugees resettled in Australia: A population-based study	186	Article	[61]
9^th^	Cognitive-behavior therapy vs exposure therapy in the treatment of PTSD in refugees	185	Article	[62]
10^th^	Psychological trauma and evidence for enhanced vulnerability for posttraumatic stress disorder through previous trauma among West Nile refugees	164	Article	[63]
11^th^	Trauma, post-migration living difficulties, and social support as predictors of psychological adjustment in resettled Sudanese refugees	160	Article	[64]
12^th^	Emerging paradigms in the mental health care of refugees	159	**Review**	[65]
13^th^	Asylum seekers and refugees in Britain: Health needs of asylum seekers and refugees	159	**Review**	[66]
14^th^	Policies of deterrence and the mental health of asylum seekers	150	Article	[67]
15^th^	Language acquisition, unemployment and depressive disorder among Southeast Asian refugees: A 10-year study	142	Article	[68]
16^th^	Mental health of immigrants and refugees	141	**Review**	[69]
17^th^	The health of immigrants and refugees in Canada	136	**Review**	[70]
18^th^	Treatment of Posttraumatic Stress Disorder by Trained Lay Counselors in an African Refugee Settlement: A Randomized Controlled Trial	124	Article	[71]
19^th^	Impact of a long asylum procedure on the prevalence of psychiatric disorders in Iraqi asylum seekers in The Netherlands	122	Article	[72]
20^th^	Comorbidity of PTSD and depression among refugee children during war conflict	121	Article	[73]

*SCR* Standard competition ranking. Equal countries were given the same ranking number, and then a gap is left in the ranking numbers

Bold = indicates a review article

### Ethnic groups

**Fig. 4:**
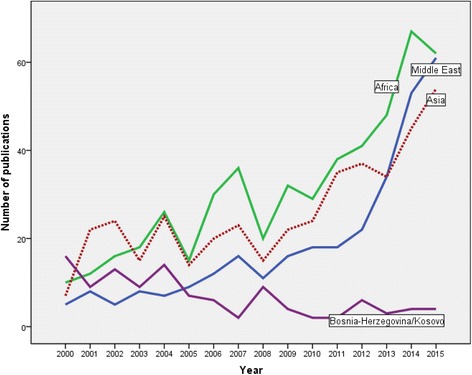
Growth of medicine – related publications on refugees, asylum seekers, and internally displaced people from Middle East, Africa, Asia, and Bosnia-Herzegovina/Kosovo

When analysis was carried out on ethnic groups mentioned in retrieved articles, publications on Somali refugees (161 articles) had the highest number of publications followed by those on Iraqi (130 articles), and Afghani (126 articles). Table 5 shows the number of publications on various ethnic groups which have been under wars and conflicts in the past two decades. Figure 4 shows the growth of publications on refugees from different world regions particularly Middle East, Asia, Africa, and Bosnia-Herzegovina/Kosovo. Data in Fig. 4 shows a steep rise on publications on refugees coming from Middle East while that on refugees from Bosnia-Herzegovina/Kosovo a declining trend.

**Table 5:** Number of medicine – related publications for selected refugee ethnicities

Ethnic group	Number of publications	Top cited article	Reference
Somalia	161	Treatment of Posttraumatic Stress Disorder by Trained Lay Counselors in an African Refugee Settlement: A Randomized Controlled Trial	[71]
Iraq	130	Impact of a long asylum procedure on the prevalence of psychiatric disorders in Iraqi asylum seekers in The Netherlands	[72]
Afghanistan	126	Physical and mental health of Afghan, Iranian and Somali asylum seekers and refugees living in the Netherlands	[74]
Bosnia- Herzegovina/Kosovo	110	Longitudinal study of psychiatric symptoms, disability, mortality, and emigration among Bosnian refugees	[59]
Sudan	94	A comparison of narrative exposure therapy, supportive counseling, and psychoeducation for treating posttraumatic stress disorder in an African refugee settlement	[57]
Syria	90	Resilience and vulnerability among refugee children of traumatized and non-traumatized parents	[75]
Burma/Mynamar/ (Karenni)	88	Karenni refugees living in Thai-Burmese border camps: Traumatic experiences, mental health outcomes, and social functioning	[76]
Pakistan	58	Mental health of displaced and refugee children resettled in low-income and middle-income countries: Risk and protective factors	[77]
Palestine	57	Comorbidity of PTSD and depression among refugee children during war conflict	[73]
Lebanon	57	Mental health and health-related quality of life: A 10-year follow-up of tortured refugees	[78]
Congo	56	Prevalence of HIV infection in conflict-affected and displaced people in seven sub-Saharan African countries: a systematic review	[79]
Ethiopi (Oromo)	54	Somali and Oromo Refugees: Correlates of Torture and Trauma History	[80]
Bhutan	51	Psychiatric disorders among tortured Bhutanese refugees in Nepal	[81]
Vietnam	49	Long-term effect of psychological trauma on the mental health of Vietnamese refugees resettled in Australia: A population-based study	[61]
China/Tibet	43	Western conceptualizations and eastern experience: A cross-cultural study of traumatic stress reactions among Tibetan refugees in India	[82]
Eritrea	30	Mental health of displaced and refugee children resettled in low-income and middle-income countries: Risk and protective factors	[77]
Burundi	20	Prevalence of HIV infection in conflict-affected and displaced people in seven sub-Saharan African countries: a systematic review	[79]
Sierra Leon	17	Prevalence of HIV infection in conflict-affected and displaced people in seven sub-Saharan African countries: a systematic review	[79]
Rwanda	14	Treatment of Posttraumatic Stress Disorder by Trained Lay Counselors in an African Refugee Settlement: A Randomized Controlled Trial	[71]

Results for each ethnic group was obtained using the following search query within the 2530 retrieved articles: TITLE(“Refugee” OR “asylum seeker” OR “displaced people”) AND TITLE-ABS-KEY(Ethnic group) AND PUBYEAR > 1999 AND PUBYEAR < 2016 AND (LIMIT-TO(SUBJAREA,“MEDI”)) AND (LIMIT-TO(SRCTYPE,“j”)) AND (EXCLUDE(DOCTYPE,“er”))

## Discussion

This study investigated the quantity and impact of medicine – related publications on refugees, asylum seekers, and IDP. Such study is important for healthcare providers and policymakers so that the basic health challenges of this category of people are met and dealt with in an appropriate way. Our study showed that the number of publications increased significantly and steeply in the past five years parallel to the increase in worldwide refugee problem. The high *h*-index of retrieved publications suggests that this subject is highly interesting to a wide category of researchers and readers in health and non-health disciplines and is being commonly cited in published articles.

The fact that most publications in this topic came from the USA, Australia, the UK, and many European countries suggests that number of refugees and asylum seekers to these countries is large and that these countries are trying to face health challenges of those people by integrating them into the health system and meet their basic health demands. The UNHCR, also known as the UN Refugee Agency, is a United Nations program that is dedicated for providing protection and support to refugees and has won two Nobel Peace Prizes, once in 1954 and again in 1981 for its humanitarian actions toward refugees all over the world. It was not surprising that the UNHCR was one of the top active organizations in publishing about health needs and challenges facing refugees. According to the UNHCR fact sheet [35], Turkey ranked first as a hosting country with 2.5 million refugees; mostly Syrians. Pakistan ranked second with 1.6 million refugees; mostly from Afghanistan. Lebanon ranked third in hosting refuges with 1.1 million refugees. Islamic Republic of Iran, Ethiopia, and Jordan ranked fourth, fifth, and sixth respectively in number of refugees being hosted in these countries. According to the same source of UNHCR fact sheet, in 2015, the USA hosted 172,000 asylum seekers, mainly from Latin America. Germany (441,900) and Sweden (173,000) had the largest number of applications for asylum in Europe. Most asylum seekers to these countries were Syrians. In 2015, 8.6 million people were newly displaced within the borders of their own countries by armed conflict, generalized violence, and human rights violations. Countries with highest numbers of IDP include Syrian Arab Republic (6.6 million), Yemen (over 2.5 million), Iraq ((808,700), Ukraine (800,000), Sudan (639,500), the Democratic Republic of the Congo (637,900), and Afghanistan (492,600).

Our study showed that top cited articles were mainly in the field of mental health. The psychological trauma of wars and violence has long term negative impact on children and adolescence. The highly cited articles were published in mental health journals or in general medicine journals with high international impact and reputation suggesting that mental health problems in specific are serious and attracting attention of international agencies, researchers, and refugees host countries.

The long history of domestic violence and war which led to the flight of millions of Somali from their country to developed countries created a large number of publications on Somali refugees. The number of publications on Syrian, Iraqi and Afghani refugees was high and matches the severity of the war and domestic conflict in these particular countries. It is expected that in the coming few years the number of publications on Syrian refugees will exceed that of the Somali due to the major health challenges encountered in the Syrian crisis. Overall, it can be noticed that publications on refugees from Middle East are matching the number of publications on African refugees. Our results showed that the number of publications on Bosnia – Herzegovina and Kosovo showed a decline with time. It should be noted that the Bosnian war started in early 1990s and ended in mid-1990s and therefore the number of publications showed a dramatic decline after signing the peace treaty in late 1995 [36]. The number of publications on refugees from Middle East showed a dramatic increase after the Syrian internal conflict in 2011. Furthermore, the Israeli invasion of Lebanon in early 1980s and Gulf war in early 1990s intensified the refugee problem in the Middle East [37–40]. The number of publications on African refugees showed a continuous increase over the study period. Several countries and regions in Africa had witnessed civil wars and great deal of killing starting from war in Somalia in early 1990s and recently in Libya [41–44]. Publications on Asian refugees are mainly due to Afghani conflict and war since late 1970s [45, 46]. Refugees from Myanmar and other south eastern Asia is a major contributor to the number of publications about Asian refugees. Internal conflicts in Myanmar (Burma) is a long one and started several decades ago and is still going [47].

The results of our study have few limitations related to the search strategy which is also typical to limitations found in several previously published bibliometric studies [21, 48–53]. For example, the title search will minimize the false positive but will create false negative results as well. Search strategy using title-abstract-keyword will definitely retrieve larger number of publications, but some of these publications will be false positive ones. Furthermore, the keywords used in our study may not be comprehensive and some authors might have used other terminology to describe refuges or IDP. These publications were missed. Finally, the study is based on Scopus database which is a perfect one, but is the largest one. Nevertheless, our study is the first to discuss this issue and to discuss research activity concerning health related issues of refugees and displaced people from a bibliometric point of view hoping that researchers in different fields will benefit from the results presented here. Furthermore, we hope that the results presented in the study will further increase the attention of healthcare providers to mental health services to refugees and asylum seekers and IDP particularly vulnerable groups of people.

## Conclusion

In conclusion, this bibliometric study showed that health – related publications on refugees, asylum seekers, and IDP was witnessed after 2011. The bulk of these publications was produced by three main countries; the USA, Australia and the UK. Publications on health of refugees appeared in high impact journal such as *Lancet* reflecting the deep concern of international health community to such human crisis. Future health related publications on this issue must be encouraged to face the increasing challenge of worldwide burden of refugees and IDP.
